# Spousal collaboration mediates the relation between self-rated health and depressive symptoms of Chinese older couples: an actor-partner interdependence approach

**DOI:** 10.1186/s12877-024-04834-4

**Published:** 2024-03-26

**Authors:** Huiying Liu, Xinyi Zhou, Mi Zhang, Bixia Chen, Jiayuan Du, Vivian Weiqun Lou

**Affiliations:** 1 Department of Sociology, Central South University, Changsha, China; 2 Department of Social Work and Social Administration, University of Hong Kong, Pokfulam, Hong Kong China; 3 Sau Po Center on Aging, 2/F, The Hong Kong Jockey Club Building for Interdisciplinary Research, University of Hong Kong, 5 Sassoon Road, Pokfulam, Hong Kong China

**Keywords:** Self-rated health, Depressive symptoms, Spousal collaboration, Older couples, Actor–partner interdependence mediation model

## Abstract

**Background:**

Dyadic coping resources have been considered a potential explanatory mechanism of spousal interdependence in health, but the mediation of spousal collaboration for the relationship between self-rated health and depressive symptoms has yet to be examined. This study aimed to investigate the within- (actor effect) and between-partner effects of self-rated health on depressive symptoms in community-dwelling older couples facing physical functioning limitations and to examine the role of spousal collaboration in mediating the actor and cross-partner effects of self-rated health on depressive symptoms.

**Method:**

Data from 185 community-dwelling older Chinese married couples were analyzed using the actor–partner interdependence mediation model (APIMeM). Couples were interviewed through trained research assistants using the 5-item common dyadic coping subscale of the Dyadic Coping Inventory (DCI), the Visual Analog Scale (VAS) of the QoL questionnaire EQ-5D and the Patient Health Questionnaire-9 (PHQ‐9).

**Results:**

Husbands’ self-rated health had an actor effect on their own depressive symptoms and a partner effect on their wives’ depressive symptoms. Wives’ self-rated health had an actor effect on their own depressive symptoms. The actor effects between self-rated health and depressive symptoms were partially mediated by their own perception of spousal collaboration. Furthermore, husbands’ self-rated health not only affects wives’ depressive symptoms directly but also indirectly by influencing wives’ perceptions of spousal collaboration.

**Discussion:**

The findings from this study underscored the importance of viewing couples’ coping processes from a dyadic and gender-specific perspective, since more (perceived) collaborative efforts have beneficial effects on both partners’ mental health outcomes.

**Supplementary Information:**

The online version contains supplementary material available at 10.1186/s12877-024-04834-4.

## Introduction

Extensive evidence has linked poor physical health conditions with deteriorated psychological well-being [1–3], including elevated depressive symptoms (i.e., clinically relevant depressive symptoms in cognitive and affective dimensions such as worthlessness, helplessness and sadness) [4]. As a global indicator of physical health conditions, self-rated health (defined as one’s subjective perception of his or her general health status) has been positively associated with elevated levels of depressive symptoms in older populations [5, 6]. Although this evidence has been largely based on studies focused on an individual’s own health [7], there is a growing appreciation of a dyadic approach to studying spousal health dynamics in later life (i.e., how health problems in one spouse influence those of the other) [8]. Empirically, both cross-sectional and longitudinal studies have examined the effects of own and spousal self-rated health on depressive symptoms [9, 10], suggesting that poor self-rated health was not only predictive of one’s own level of depressive symptoms but may also lead to an increase in depressive symptoms of the respective spouse [11].

Despite the emerging evidence on spousal interdependence, little is known about the within-couple interrelations between self-rated health and depressive symptoms in the context of physical functioning limitations (i.e., defined as the state between physical impairment and disability according to the disablement process model) [12, 13]. Indeed, both spouses having physical functioning limitations (often measured by self-reported incapability to perform physical tasks such as walking, lifting and carrying) is an increasingly prevalent phenomenon in community-dwelling older adults [14]. It is especially important to consider both spouses’ health with possible interrelations among these couples for at least two reasons. First, when both spouses are facing the age-related challenged of functional limitations, the arising needs for not only managing one’s own functional limitations but also helping with the partner’s daily task can produce considerable psychological stress, which may put these spouses at higher risk of unfavorable subjective experiences [15–17]. Second, spouses facing physical functioning limitations typically share similar environmental and risk factors (e.g., activity restrictions, negotiation on housework allocation), leading to negative emotional contagion, and within-couple interactions may also increase the tendency for ratings of poor self-rated health in one partner to affect those in the other [18]. Therefore, the current study adopts a dyadic approach to investigate spousal interdependence between self-rated health and depressive symptoms in older couples wherein both spouses facing physical functioning limitations. Using independent data collected separately from husbands and wives, we are interested in whether the impact of self-rated health on increased depressive symptoms occurred not only at the individual level but also at the couple level.

To understand spousal health interdependence in the context of physical functioning limitations, we incorporate the Vulnerability Stress and Adaptation (VSA) model and recent conceptualizations of dyadic coping, including the Systematic Transactional Model [19], to develop our research framework. These theoretical works posit that aging-related health decline (e.g., diseases, functional limitations) represents a common stressor that affects both marital partners simultaneously, which requires them to engage in dyadic coping strategies (in addition to individual coping effort) [20]. As a positive dyadic coping strategy, spousal collaboration refers to the collective efforts that spouses use to manage their common stress (e.g., discussing problem solutions, working together to solve everyday tasks), which plays an important role in shaping spousal interrelations in health appraisals and outcomes [21, 22]. In our research framework, physical functioning limitation is such a chronic stressor facing each spouse that initiates a dyadic coping process in which spousal collaboration serves as a potential mechanism explaining how each partner’s perceived health resource would be linked to health outcomes of the self and the partner (see Supplementary Fig. 1 for a visual illustration of our research framework).

Specifically, we build on the Systematic Transactional Model and its supporting research to develop research hypotheses concerning the role of spousal collaboration in the individual and dyadic effects of self-rated health on depressive symptoms. One spouse’s appraisal of individual coping resources (e.g., self-rated health) has been suggested as influential for his or her engagement in collaborative activities [23], which can subsequently provide a range of health benefits (e.g., enhanced relationship satisfaction and psychological well-being) [24]. In older spouses facing functioning limitations, better self-rated health may serve as a necessary health resource that activates one’s engagement in spousal collaboration, which in turn buffers negative psychological outcomes (e.g., reducing the level of depressive symptoms). Thus, we expect spousal collaboration to mediate the link between one’s self-rated health and depressive symptoms (actor effect). On the other hand, there is limited evidence on the role of spousal collaboration in influencing the cross-partner relationship between health appraisals and outcomes (dyadic effect), and to our knowledge, no study has examined whether spousal collaboration mediates the dyadic effects of self-rated health on depressive symptoms. Notably, evidence of gender differences in couple-based collaboration with determinants is emerging, suggesting a general pattern that females were more sensitive to their spouse’s subjective experiences, such as appraisals of health problems, self-disclosure and emotional expression, and are often more strongly influenced by their spouses than males [25–27]. Accordingly, we expect to observe gender differences in the role of spousal collaboration in the dyadic effects we examined; that is, self-rated health would be more predictive of the spouse’s level of collaboration and depressive symptoms among husbands (than among wives).

The present study aims to examine the relations between self-rated health and depressive symptoms at the individual and dyadic levels and to investigate the role of spousal collaboration in mediating the individual and dyadic effects of self-rated health on depressive symptoms. We focused on a sample of community-dwelling older married couples wherein both spouses had physical functioning limitations (an age-related common stressor that affects both spouses and requires their collaborative efforts). Building on the theoretical work and the relevant literature, we tested the following hypotheses.

### Hypothesis 1

Self-rated health is negatively associated with the spouse’s own level of depressive symptoms (actor direct effect) and the respective partner’s level of depressive symptoms (partner direct effect).

### Hypothesis 2

Spousal collaboration mediates the negative association between one’s self-rated health and his or her own level of depressive symptoms (actor indirect effect).

### Hypothesis 3

Spousal collaboration mediates the negative association between one’s self-rated health and the respective partner’s level of depressive symptoms (partner indirect effect).

## Method

### Participants and procedure

The data from the present study were collected from January 2020 to March 2023 at two sites: Hong Kong City and Changsha City (the capital city of Hunan Province located in South-Central China). The purposeful quota sampling method was used to recruit eligible couples. The inclusion criteria were as follows: (1) heterosexual married couples living in the same household; (2) one or both partners aged 60 or above; and (3) both partners reporting one or more physical functioning limitations, measured by self-report questions asking each spouse’s perceived difficulties in performing the following eight tasks, including jogging 1 km, walking 1 km outdoors, bending, stooping or crouching, stretching arms up along shoulders, carrying a weight of 10 pounds, picking up a coin from the table, getting up from a chair and climbing several flights of stairs in a row. The exclusion criteria were as follows: (1) one or both partners having severe visual or hearing deficits; (2) reporting severe physical disability (e.g., perceived difficulties in feeding, bathing, grooming, getting dressed, bowel control, bladder control, using the toilet, chair/bed transfer, mobility and climbing stairs.); (3) being diagnosed by a clinician as having cognitive diseases (e.g., dementia) or depression.

We adopted a three-stage recruitment approach from the district-community-individual, with some differences in practice at the two sites. At site 1, four of all eight urban districts were randomly selected, and the full list of community centers was obtained from each district. The project investigators selected the community centers in two steps. First, an initial screening of all the listed community centers was based on whether (1) the area served by the community center is highly aging and the community center has specialized facilities and staff for the provision of geriatric services and (2) the geriatric service staff at the community centers are in close contact with and familiar with the older couples in their districts so that they can provide a list of potential participants for the present study. Second, the project investigators made phone calls with the executive director of selected centers to confirm their collaboration willingness and capacity to recruit older couples and implement data collection during the COVID-19 epidemic. The four selected centers (including two District Elderly Community Centers (DECC) and two Neighborhood Elderly Centers (NEC) were responsible for providing a list of potential eligible couples and helping us make initial contacts. At site 2, four of six urban administrative districts were selected, followed by randomly selecting one street office from each district and selecting one local resident committee from each street office. Each resident committee provided the referral list of potential eligible couples and referred a worker from the Office of Aging Work or a Party-masses Work Department to help with making appointments with these couples. At both sites, the trained interviewers conducted the 10-minute brief screening for each referred couple to confirm their study eligibility during the first appointment. Once eligible, the researcher asked about the couple’s willingness to participate and obtained informed consent. The eligible couple was then invited to complete the 45-minute geriatric assessment questionnaire (covering information on sociodemographic features, health history, and perceptions of spousal coping, etc.). To avoid mutual influence between spouses, this assessment was conducted by two interviewers for the husband and wife separately (e.g., in two separate rooms or spaces). After completing this baseline assessment, each spouse was assigned a paper booklet and instructed to complete two recordings per day during the following 20-day EMA data collection period. Since our data collection was conducted during the COVID-19 epidemic, all the interviews were arranged to accommodate participants’ convenience, either at the participant’s home or meeting rooms of the local resident committee office (or community centers). To achieve the expected sample size, the snowball sampling method was used at later recruitment stages at both sites (*n* = 47 dyads, 25.4%). We asked the participants to recommend potential participants living in the same community and help to ask about their willingness to participate. Appointments were then arranged for those couples showing interest in participation following the same procedure described above.

The final sample included 185 married couples (site 1: 77 dyads, site 2: 108 dyads, *N* = 370 participants). See Table 1 for more information about the demographic characteristics of the study sample. The husbands’ mean age was 77.06 (SD = 7.66; range: 60–96), and the women’s mean age was 73.76 years (SD = 7.32; range: 53–91). On average, the marital duration was 47.76 years (SD = 10.18). A total of 12.4% of women and 4.3% of men were uneducated; 73.0% of women and 70.8% of men had finished middle school; and 14.6% of women and 24.9% of men had a bachelor’s degree. A total of 96.2% of women and 96.8% of men were not currently working. Couples had on average 1.89 children (SD = 1.10; range: 0–6 children). The percentages of women and men with more than 2 chronic diseases were 57.7% and 50.5%, respectively.

**Table 1:** Characteristics and independent sample T tests of the variables

Characteristics		Husbands	Wives	P value
		M(SD)	M(SD)	
Self-rated health		74.75(15.51)	73.44(15.40)	0.414
Spousal Collaboration		18.69(4.50)	18.90(4.94)	0.676
Depressive symptoms		12.08(3.47)	13.13(3.78)	0.006*
Age		77.06(7.66)	73.76(7.32)	0.000*
Marital duration		47.76(10.18)		-
		*Count(%)*	*Count(%)*	
Education level				0.002*
Illiterate		8(4.3)	23(12.4)	
Educated		177(95.7)	162(87.6)	
Employment status				1.000
Not working		179(96.8)	178(96.2)	
Currently working		6(3.2)	7(3.8)	
Number of Chronic disease				0.014*
0		8(4.4)	20(11.0)	
1		34(18.7)	22(12.1)	
2		48(26.4)	35(19.2)	
Above 2		92(50.5)	105(57.7)	
Number of children				-
0		7(3.8)		
1		68(36.8)		
2		71(38.4)		
Above 2		39(21.1)		
Physical Functioning Limitations				1.000
1		60(32.4)	57(30.8)	
2		43(23.2)	44(23.8)	
Above 2		82(44.4)	84(45.4)	

*Note* Independent sample T tests for continuous variables and chi-square tests for classified variables were conducted. Significant coefficients are in bold (* *p*<.05; two-tailed)

### Measures

Self-rated health. The Visual Analog Scale (VAS) of the QoL questionnaire EQ-5D was used [28]. This VAS ranges from 0 (worst possible health) to 100 (best possible health). Respondents were asked to assess their present health using this scale. Scores were coded such that higher scores reflected better health. This measure is often used in survey research and has been associated with objective physical health status [29].

Depressive symptoms. The Patient Health Questionnaire–9 (PHQ-9) [30] was used to examine depressive symptoms. The PHQ-9 scale is composed of nine items relating to symptoms of depression as defined by the DSM-IV. Each of the items is scored from 0 (not at all) to 3 (almost every day), resulting in a maximum total score of 27, with higher scores representing more severe depression. The internal consistency of the PHQ-9 in the current study was α = 0.757 for wives and α = 0.719 for husbands.

Spousal Collaboration. The subscale of the validated Chinese version of the Dyadic Coping Inventory (DCI) [31] was used to measure spousal collaborative behaviors. The Dyadic Coping Inventory (DCI) is a widely used [32] self-report questionnaire developed by Bodenmann (2008) to assess partners’ stress expression and dyadic coping behaviors, which include the communication of one partner’s stress, supportive dyadic coping, delegated dyadic coping, negative dyadic coping, and common or joint dyadic coping. For the present study, we assessed couples’ perception of the usage of common dyadic coping through a total of 5 items. Husbands and wives indicated how often they, as a couple, engaged in a series of activities to deal with stress. Sample items are “We try to cope with the problem together and search for and “We help one another to put the problem in perspective and see it in a new light.” Couples rated how often they (self) and their partners (partner) engaged in spousal collaborative strategies on a 5-point Likert scale (1 = not at all/very rarely to 5 = very often). The internal consistency of the common dyadic coping scale in the current study was α =.86 for wives and α =.82 for husbands.

### Analytic strategy

We used the extended version of the actor–partner interdependence model (APIM) [33]—APIMeM to examine spillover and crossover processes and to test for mediating effects. This approach provides a method to simultaneously examine how a person’s own health and the health of his or her spouse are related to depressive symptoms [34, 35]. It allowed us to include mediation variables in the model, to control for the interdependence of dyadic data and to achieve separate estimates for actor and partner effects. APIMeM models were estimated using MPlus version 8.0. SPSS 26 was used to compute descriptive statistics and to account for the nonmoral distribution. Due to the nonmoral data, we used the MLR estimator (maximum likelihood estimation with robust standard of errors) for a robust estimation to obtain standardized coefficients. Good model fit was interpreted according to Hu and Bentler’s (1999) recommendations: (1) a small and nonsignificant chi-square test; (2) root mean square error of approximation (RMSEA) less than or equal to 0.06; (3) standardized root mean squared residual (SRMR) less than 0.08; and (4) comparative fit index (CFI) and Tucker‒Lewis index (TLI) greater than or equal to 0.9. On the basis of random sampling from the dataset, bootstrapping produces slightly different estimates of the indirect effect and its standard error, as well as the upper and lower bounds of confidence intervals from run to run. There is no agreement on the optimal number of bootstrap samples, but it is generally accepted that the greater the number of bootstrap samples taken, the greater the stability of the CI bounds will be over consecutive runs of the program [36]. Considering statistical power and the stability of the CI bounds, separate bootstrap analyses with 7,000 samples and bootstrapped-corrected 95% confidence interval test statistics were used in the final analysis. The research steps were as follows: (1) first judge whether it is a distinguishable dyad; (2) If it is a distinguishable dyad, a corrected saturation model was constructed to estimate the actor effect and partner effect, and then the total effect is calculated, direct effect and indirect effect; and (3) Confidence intervals of each effect value were obtained by the bootstrap method. After correcting the saturation model to estimate the actor effect and the partner effect, the indirect effect and the total effect were obtained by adding the corresponding effect values. According to Ledermann et al. [37], a sample size of approximately 93 to 241 dyads was recommended in the actor-partner interdependence model (APIM), and a sample size of approximately 120 dyads was needed for a good-powered mediation in the actor-partner interdependence mediation model (APIMeM). Meanwhile, given the use of structural equation modeling with observed variables in our dyadic analyses, we followed sample size recommendations in multiple regression analyses, as suggested by Kenny and Cook [38]. A power analysis conducted by G*Power 3.1.9.7 determined that, with twelve predictors (including two independent variables and ten possible control variables), a minimum of 120 dyads were needed to achieve a medium mediation effect (*f*^2^ = 0.16) in the APIMeM with a significance level of 0.05 and a power of 0.8.

## Results

### Bivariate correlations

Table 2 presents intercorrelations for all study variables. In general, the scores for the study variables were moderate for self-rated health and depressive symptoms, and wives reported significantly higher scores on depressive symptoms than husbands, t (368) = 0.2.781, *p* <.05. Couples also reported above-average levels of spousal collaborative behaviors. As shown in Table 2, the self-rated health of husbands and wives was positively correlated with their own spousal collaboration and negatively correlated with their own depressive symptoms. Table 2 also reveals the between-partner correlations: we found significant positive correlations between the two partners’ levels of spousal collaboration (see Supplementary Table 2 for effect-size estimation).

**Table 2:** Correlations among study variables

Variables	1	2	3	4	5	6
1. husbands’ Self-rated health	1.000	0.111	0.180*	0.181*	− 0.353**	− 0.141
2. wives’ Self-rated health		1.000	− 0.003	0.210**	− 0.024	− 0.452**
3. husbands’ Spousal collaboration			1.000	0.232**	− 0.318**	− 0.031
4. wives’ Spousal collaboration				1.000	− 0.040	− 0.198**
5. husbands’ Depressive symptoms					1.000	0.137
6. wives’ Depressive symptoms						1.000

*Note* Range for self-rated health: 0–100; range for depressive symptoms: 0–27; and range for spousal collaboration: 5–25. Significant correlations are in bold, * *p* <.05, ** *p* <.01, (two-tailed)

**Fig. 1:**
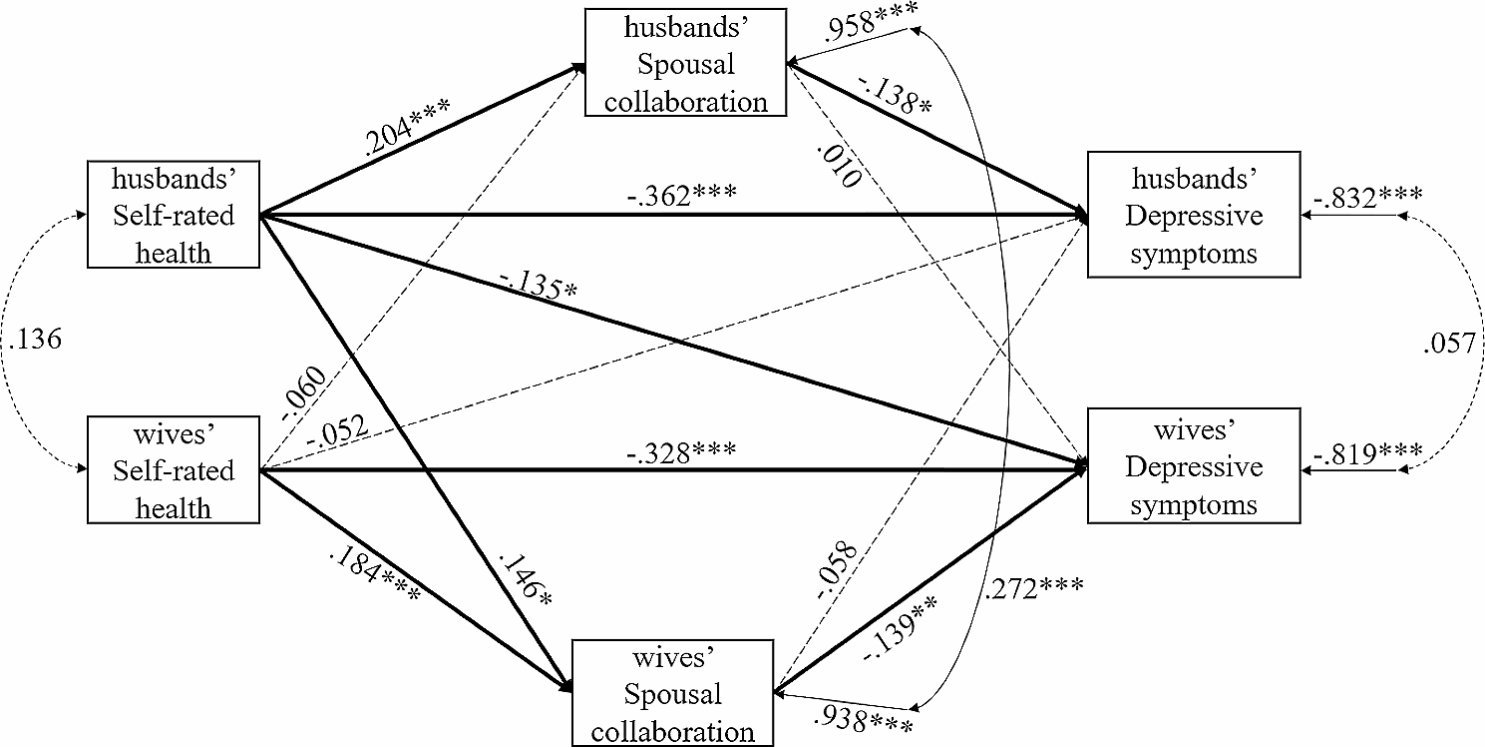
APIMeM testing spousal collaboration as a mediating variable in the relationship between self-rated health and depressive symptoms. *Note* The standardized coefficients are reported in Fig. 1. All results were from modeling with the MLR estimator, but for the 95% CI of the indirect effect, the ML estimator was used in Mplus 8. Complete arrows represent significant direct effects, and dotted arrows represent nonsignificant effects. * *p* <.05, **p <.01, ***p <.001 (two-tailed)

The APIMeM is illustrated in Fig. 1. The model showed a satisfactory fit: χ^2^ (3) = 1.159, *p* =.324, RMSEA = 0.029, SRMR = 0.023, CFI = 0.995, TLI = 0.977. Considering that χ^2^ is sensitive to sample size, we applied the rule that χ^2^/df should be smaller than 3.

### Direct effects: self-rated health, spousal collaboration, and depressive symptoms

Table 3 presents the standardized coefficients and bootstrapped-corrected 95% CI of the direct effects and indirect effects for the APIMeM. The results indicated that husbands’ SRH had a significant direct effect on their own spousal collaboration, and wives’ SRH also had a significant direct effect on their own spousal collaboration (β_Wives_ = 0.204 with the CI is [0.099,0.306], β_Husbands_ = 0.184 with the CI is [0.089,0.273]). In terms of the partner effect, the association between husbands’ self-rated health and wives’ spousal collaboration was significant (β_Husbands_ = 0.146 with the CI is [0.008,0.273]). The actor direct paths from self-rated health to depressive symptoms were statistically significant in the structural model (β_Wives_= -0.328 with the CI is [-0.449, -0.213], β_Husbands_= -0.362 with the CI is [-0.486, -0.245]), and there was a significant partner direct effect between husbands’ self-rated health and wives’ depressive symptoms (β_Husbands_= -0.135 with the CI is [-0.268, -0.002]). This finding was consistent with previous empirical studies showing an obvious negative correlation between self-rated health and depressive symptoms. The actor direct paths from spousal collaboration to depressive symptoms were statistically significant, showing that significant effects, namely, husbands’ and wives’ spousal collaboration, were significantly negatively related to their own depressive symptoms (β_Wives_=-0.139 with the CI is [-0.238, -0.039], β_Husbands_= -0.138 with the CI is [-0.254, -0.036]).

**Table 3:** Direct effects and indirect effects for the APIMeM

Effect	Estimate	SE	95% CI lower bound	95% CI upper bound
husbands’ Self-rated health → husbands’ Depressive symptoms				
Total	**-0.382**	0.059	**-0.495**	**-0.267**
Total indirect	-0.020	0.016	-0.054	0.011
Indirect (husbands’ Spousal collaboration)	**-0.028**	0.014	**-0.063**	**-0.008**
Indirect (wives’ Spousal collaboration)	0.008	0.012	-0.007	0.044
Direct	**-0.362**	0.063	**-0.486**	**-0.245**
wives’ Self-rated health → wives’ Depressive symptoms				
Total	**-0.354**	0.058	**-0.471**	**-0.241**
Total indirect	**-0.026**	0.013	**-0.055**	**-0.005**
Indirect (husbands’ Spousal collaboration)	-0.001	0.006	-0.018	0.010
Indirect (wives’ Spousal collaboration)	**-0.026**	0.012	**-0.055**	**-0.008**
Direct	**-0.328**	0.061	**-0.449**	**-0.213**
husbands’ Self-rated health → wives’ Depressive symptoms				
Total	**-0.153**	0.069	**-0.291**	**-0.019**
Total indirect	-0.018	0.021	-0.065	0.019
Indirect (husbands’ Spousal collaboration)	0.002	0.015	-0.028	0.030
Indirect (wives’ Spousal collaboration)	**-0.020**	0.013	**-0.053**	**-0.002**
Direct	**-0.135**	0.068	**-0.268**	**-0.002**
wives’ Self-rated health → husbands’ Depressive symptoms				
Total	-0.033	0.088	-0.211	0.132
Total indirect	0.019	0.017	-0.011	0.057
Indirect (husbands’ Spousal collaboration)	0.008	0.011	-0.007	0.036
Indirect (wives’ Spousal collaboration)	0.011	0.013	-0.012	0.042
Direct	-0.052	0.086	-0.229	0.110

*Note* SE standard error, CI confidence interval. The standardized coefficients are reported in Table 3. Significant coefficients are in bold (The 95% confidence interval did not include 0)

### Indirect effects: the mediating role of spousal collaboration

For the main aspects we wanted to explore, we assumed that spousal collaboration explained the influence of self-rated health on depressive symptoms. The findings showed that spousal collaboration mediated the actor effects of self-rated health on depressive symptoms. Wives’ self-rated health had a significant indirect effect on their own depressive symptoms through their own spousal collaboration (β_Wives_=-0.026 with the CI is [-0.055, -0.005]). Husbands’ self-rated health also had a significant indirect effect on their own depressive symptoms through their own spousal collaboration (β_Husbands_ =-0.028 with the CI is [-0.063, -0.008]).

In addition to indirect actor–actor effects, we found an indirect actor–partner effect: husbands’ self-rated health had an indirect effect on wives’ depressive symptoms through wives’ spousal collaboration (β_Wives_=-0.020 with the CI is [-0.053, -0.002]). However, another indirect actor–partner effect between wives’ self-rated health and husbands’ depressive symptoms through husbands’ spousal collaboration was not found (β_Wives_ = 0.008 with the CI is [-0.007,0.036]).

We conducted a series of sensitivity analyses, including (1) rerunning the model after removing the 12 pairs of couples in the sample who were employed full-time; (2) adding age, number of children, education level and physical limitations as control variables and rerunning the model; and (3) rerunning the model by including the sample site as control variables to account for the differences in the sociocultural backgrounds of our sample sources. The results from the sensitivity analysis above were generally consistent with our main findings except that the direct effect between husbands’ self-rated health and wives’ depressive symptoms could be minimal and negligible which was not robust regardless of the resampling sample size and the p values across models. (please refer to Additional file 1 for details).

## Discussion

This study adopted a dyadic approach to investigate the role of spousal collaboration in influencing the individual and dyadic links between self-rated health and depressive symptoms in a sample of community-dwelling old spouses with physical functioning limitations. The actor–partner analyses indicated that poor self-rated health was not only associated with one’s own level of depressive symptoms but was also predictive of that of the respective spouse. Spousal collaboration significantly mediated the individual links between self-rated health and depressive symptoms for both husbands and wives. The cross-partner effect was observed only in wives, that is, wives’ spousal collaboration was positively associated with husbands’ better self-rated health, which in turn buffered wives’ depressive symptoms.

Extending research on the links between physical health and depressive symptoms that were mostly established at the individual level [39, 40], our study revealed significant dyadic effects of spousal self-rated health on depressive symptoms among older couples facing physical functioning limitations. We found that one’s self-rated health (how one views his or her health) not only matters for himself or herself but also significantly influences the respective spouse’s psychological well-being. As the sampled older couples faced the challenge of both spouses having functioning limitations, which were often accompanied by considerable psychological stress due to arising needs for managing daily tasks and care responsibilities [11], spouses may tend to internalize the partner’s health problems and experience more negative feelings such as grief and anxiety [39–41]. Therefore, more attention should be given to the assessment of each spouse’s self-perceptions of health with possible improvement strategies, which can be valuable for understanding the transmission and maintenance of depressive symptoms [42], especially for older spouses who have internalized problems when facing long-term health declining challenges such as physical functioning limitations [43, 44].

Our study was the first to examine the role of spousal collaboration in the association between self-rated health and depression symptoms at both the individual level and the dyadic level. Confirming our expectation, spousal collaboration exerted a significant actor-mediating effect for both husbands and wives (at the individual level). That is, better self-rated health was associated with one’s own higher level of spousal collaboration, which in turn was associated with his or her fewer depressive symptoms. These findings were consistent with theoretical and empirical works on dyadic coping, indicating that spousal collaboration may serve as a protector against unfavorable psychological outcomes by enhancing partners’ perception that they are “together” and promoting a sense of “normalcy” or cohesion within the marital relationship [45]. By providing the first evidence on the effectiveness of spousal collaboration in buffering the negative impact of health problems on depressive symptoms, our study extended current conceptualizations of dyadic coping into a largely overlooked group of older couples wherein both spouses facing physical functioning limitations [46].

Notably, our findings pointed out a clear gender difference in the mediating role of spousal collaboration in the cross-partner relationship between self-rated health and depressive symptoms. We found that the wife’s level of spousal collaboration was positively associated with their husband’s better self-rated health and in turn buffered against an increase in their own level of depressive symptoms, while a similar effect was not found among husbands. In line with prior research suggesting that wives were more responsive to their partners’ health problems [27], we speculated that these cross-partner influences observed among wives but not among husbands might be relevant to gender differences in relationship orientation and emotional contagion. It is also possible that wives tended to show a higher level of sensitivity to the qualitative aspects of their partner’s health, especially those negative subjective experiences surrounding health problems [47, 48]. Future research investigating possible gender differences in psychosocial determinists of spousal collaboration (e.g., motivating factors) is warranted.

Our findings can provide insights into clinical practices with respect to the following aspects. First, more attention should be given to the transmission of poor health appraisals and depressive symptoms in older couples facing the challenge of physical functional limitations. While often falling outside the scope of current family-based health services and programs, this group of older couples has been found to be especially susceptible to the influence of their spouses. Second, the enhancement of spousal collaboration should be incorporated into current family-based health interventions and programs targeting older Chinese married couples, especially couples facing physical health problems. These interventions would not only help enhance their performance in collaborative tasks but also provide benefits for their psychological well-being. Third, it is also important for community health workers and practitioners to shift from an individual-based paradigm to a couple-based paradigm by treating the couple as a cohesive unit throughout the different stages of program implementation. For example, multidimensional geriatric assessment should be conducted at the couple level to collect information on the health vulnerabilities and resources of each spouse. In addition, mental health practitioners working on couple-based therapy and consultation may need to differentiate actor and partner effects of objective health indicators and psychological resources on depressive symptoms [49] and gender disparities in risk and protective factors for mental health.

This study had two major strengths. First, our study was the first to examine spousal interdependence in self-rated health and depressive symptoms in older couples wherein both spouses facing physical functional decline, an increasingly prevalent but largely overlooked group of community-dwelling older people in both research and practices targeting spousal health interdependence. Second, our study took a dyadic approach to examine both the actor and cross-partner effects of spousal collaboration on the relationship between self-rated health and depressive symptoms. Using independent data collected from each spouse from the same couple, our study provided the first evidence that spousal collaboration mediated the cross-partner effect of self-rated health on depressive symptoms among wives. Nevertheless, this study also has several limitations. First, self-reported data have inherent methodological limitations (e.g., social desirability, common method variance problem). The measures used in our study are brief, self-report measures, and the possibilities of under- or overreporting cannot be evaluated. Second, the cross-sectional and correlational research design does not allow real causal inferences about relationships among variables to be made. Our findings need to be cross-validated and replicated using dyadic longitudinal data to help clarify the directions of the effects in the health-depressive symptoms links. For example, multiple assessments over time would allow researchers to determine the trajectory of change in health and depressive symptoms and assess a possible causal link between changes in these variables.

## Conclusion

Overall, our study identified spousal collaboration as a mediating mechanism underlying spousal health interrelations between self-rated health and depression symptoms. For both husbands and wives, there was a negative association between their respective self-rated health and depressive symptoms, which was mediated by spousal collaborative behaviors. Wives’ spousal dyadic behaviors and depressive symptoms were more likely to be influenced by their husbands. The findings of our study extended current conceptualizations of dyadic coping to a rarely examined context of both spouses facing physical functioning limitations and can inform future development of couples-based health interventions by underscoring the importance of incorporating the enhancement of spousal collaboration as a key element.

### Electronic supplementary material

Below is the link to the electronic supplementary material.

Supplementary Material 1

## Data Availability

The datasets used and/or analyzed during the current study are available from the corresponding author upon reasonable request.

## References

[1] Thompson WW, Zack MM, Krahn GL, Andresen EM, Barile JP (2012). Health-related quality of life among older adults with and without functional limitations. Am J Public Health.

[2] Hirsch JK, Walker KL, Chang EC, Lyness JM (2012). Illness burden and symptoms of anxiety in older adults: optimism and pessimism as moderators. Int Psychogeriatr.

[3] Paukert AL, Pettit JW, Kunik ME, Wilson N, Novy DM, Rhoades HM, Greisinger AJ, Wehmanen OA, Stanley MA (2010). The roles of social support and self-efficacy in physical health’s impact on depressive and anxiety symptoms in older adults. J Clin Psychol Med Settings.

[4] Shorey S, Ng ED, Wong CH (2022). Global prevalence of depression and elevated depressive symptoms among adolescents: a systematic review and meta-analysis. Br J Clin Psychol.

[5] Benyamini Y (2011). Why does self-rated health predict mortality? An update on current knowledge and a research agenda for psychologists. Psychol Health.

[6] Peleg S, Nudelman G (2021). Associations between self-rated health and depressive symptoms among older adults: does age matter?. Soc Sci Med.

[7] Wilson SJ, Novak JR, Yorgason JB, Martire LM, Lyons KS. New Opportunities for Advancing Dyadic Health Science in Gerontology. Gerontologist 2022, gnac187. 10.1093/geront/gnac187 PMC10733121 36534908

[8] Novak JR, Wilson SJ, Ermer AE, Harper JM (2023). Aging together: dyadic profiles of older couples’ marital quality, psychological well-being, and physical health. J Social Personal Relationships.

[9] Pruchno R, Wilson-Genderson M, Cartwright F (2009). Self-rated health and depressive symptoms in patients with end-stage renal disease and their spouses: a longitudinal dyadic analysis of late-life marriages. Journals Gerontology: Ser B.

[10] Hoppmann; Gerstorf D (2009). Spousal interrelations in old age–a mini-review. Gerontology.

[11] Valle G, Weeks JA, Taylor MG, Eberstein IW (2013). Mental and physical health consequences of spousal health shocks among older adults. J Aging Health.

[12] Simonsick EM, Kasper JD, Guralnik JM, Bandeen-Roche K, Ferrucci L, Hirsch R, Leveille S, Rantanen T, Fried LP (2001). Severity of upper and lower extremity functional limitation: scale development and validation with self-report and performance-based measures of physical function. Journals Gerontol Ser B: Psychol Sci Social Sci.

[13] Verbrugge LM. Revisiting the disablement process. Int Handb Health Expectancies 2020, 275–85.

[14] Liu H, Li Y, Wang Y, Morrow-Howell N, Lou VWQ, Shen H-W (2021). Within-couple dissimilarities in functional impairment as determinants of spousal care arrangement among older married couples. Res Nurs Health.

[15] Angner E, Ghandhi J, Williams Purvis K, Amante D, Allison J (2013). Daily functioning, health status, and happiness in older adults. J Happiness Stud.

[16] Galenkamp H, Deeg DJ, Huisman M, Hervonen A, Braam AW, Jylhä M (2013). Is self-rated health still sensitive for changes in disease and functioning among nonagenarians?. Journals Gerontol Ser B: Psychol Sci Social Sci.

[17] Caramenti M, Castiglioni I (2022). Determinants of Self-Perceived Health: the importance of Physical Well-being but also of Mental Health and Cognitive Functioning. Behav Sci.

[18] Zhao X, Li D, Zhang Q, Liu H (2022). Spousal concordance in frailty predicting mental and functional health decline: a four-year follow‐up study of older couples in urban and rural China. J Clin Nurs.

[19] Badr H, Acitelli LK (2017). Re-thinking dyadic coping in the context of chronic illness. Curr Opin Psychol.

[20] Badr H, Ahmad Z. Couple-relationships and cancer adaptation. Wiley Encyclopedia Health Psychol 2020, 81–8.

[21] Bodenmann G (2008). Dyadic coping and the significance of this concept for prevention and therapy. Z für Gesundheitspsychologie.

[22] Berg CA, Upchurch R (2007). A developmental-contextual model of couples coping with chronic illness across the adult life span. Psychol Bull.

[23] Bodenmann G, Randall AK, Falconier MK. Coping in couples: the systemic transactional model (STM). In *Couples coping with stress*, Routledge: 2016; pp 5–22.

[24] Falconier MK, Kuhn R (2019). Dyadic coping in couples: a conceptual integration and a review of the empirical literature. Front Psychol.

[25] Hagedoorn M, Sanderman R, Ranchor AV, Brilman EI, Kempen GI, Ormel J (2001). Chronic disease in elderly couples: are women more responsive to their spouses’ health condition than men?. J Psychosom Res.

[26] Muramatsu Y, Takagi K, Suzuki T, Dhungel B, Tsuchiya A, Wada K (2021). Does poor spousal health negatively affect own health among elderly retired Japanese couples? A 1-year follow-up study. SSM-Population Health.

[27] Min J, Yorgason JB, Fast J, Chudyk A (2020). The impact of spouse’s illness on depressive symptoms: the roles of spousal caregiving and marital satisfaction. Journals Gerontology: Ser B.

[28] Wang Q, Liu X, Zhu M, Pang H, Kang L, Zeng P, Ge N, Qu X, Chen W, Hong X (2020). Factors associated with health-related quality of life in community‐dwelling elderly people in China. Geriatr Gerontol Int.

[29] Wu S, Wang R, Zhao Y, Ma X, Wu M, Yan X, He J (2013). The relationship between self-rated health and objective health status: a population-based study. BMC Public Health.

[30] Levis B, Benedetti A, Thombs BD. Accuracy of Patient Health Questionnaire-9 (PHQ-9) for screening to detect major depression: individual participant data meta-analysis. *bmj* 2019, *365*. 10.1136/bmj.l1476 PMC6454318 30967483

[31] Xu F, Hilpert P, Randall AK, Li Q, Bodenmann G (2016). Validation of the Dyadic coping inventory with Chinese couples: factorial structure, measurement invariance, and construct validity. Psychol Assess.

[32] Shujja S, Adil A, Randall AK, Bodenmann G, Malik F (2020). Psychometric properties and validity of Dyadic Coping Inventory-Urdu Version for use in Pakistan. Interpersona: Int J Personal Relationships.

[33] Ledermann T, Macho S, Kenny DA (2011). Assessing mediation in Dyadic Data using the actor-Partner Interdependence Model. Struct Equation Modeling: Multidisciplinary J.

[34] Bodenmann G, Ledermann T, Bradbury TN (2007). Stress, sex, and satisfaction in marriage. Personal Relationships.

[35] Kenny DA, Kashy DA, Cook WL. *Dyadic data analysis*. Guilford Publications: 2020.

[36] Preacher KJ, Hayes AF (2004). SPSS and SAS procedures for estimating indirect effects in simple mediation models. Behav Res Methods Instruments Computers.

[37] Ledermann T, Rudaz M, Wu Q, Cui M (2022). Determine power and sample size for the simple and mediation actor–Partner Interdependence Model. Fam Relat.

[38] Kenny DA, Cook W (1999). Partner effects in relationship research: conceptual issues, analytic difficulties, and illustrations. Personal Relationships.

[39] He M, Ma J, Ren Z, Zhou G, Gong P, Liu M, Yang X, Xiong W, Wang Q, Liu H (2019). Association between activities of daily living disability and depression symptoms of middle-aged and older Chinese adults and their spouses: a community based study. J Affect Disord.

[40] Monserud MA, Peek MK (2014). Functional limitations and depressive symptoms: a longitudinal analysis of older Mexican American couples. Journals Gerontol Ser B: Psychol Sci Social Sci.

[41] Hoppmann CA, Gerstorf D, Hibbert A (2011). Spousal associations between functional limitation and depressive symptom trajectories: longitudinal findings from the study of Asset and Health dynamics among the Oldest Old (AHEAD). Health Psychol.

[42] Kim Y, Kim K, Boerner K, Han G (2018). Aging together: self-perceptions of aging and family experiences among Korean baby boomer couples. Gerontologist.

[43] Wang J, Wang Q, Hou X-Y, Chen S, Guo Z, Du W, Fan L (2021). Spousal concordance in the Development of Functional limitations among married adults in China. JAMA Netw open.

[44] Polenick CA, Birditt KS, Turkelson A, Kales HC (2020). Individual-level and couple-level discordant chronic conditions: longitudinal links to functional disability. Ann Behav Med.

[45] Watts KJ, Sherman KA, Mireskandari S, Meiser B, Taylor A, Tucker K (2011). Predictors of relationship adjustment among couples coping with a high risk of developing breast/ovarian cancer. Psychol Health.

[46] Lou VW, Lu N, Xu L, Chi I (2013). Grandparent-grandchild family capital and self-rated health of older rural Chinese adults: the role of the grandparent-parent relationship. J Gerontol B Psychol Sci Soc Sci.

[47] Schmitt M, Kliegel M, Shapiro A (2007). Marital interaction in middle and old age: a predictor of marital satisfaction?. Int J Aging Hum Dev.

[48] Zhao X, Zhang Q, Xu H, Li X, Lou VW, Liu H. Unmet needs and depression among spousal caregivers: the mediating role of marital satisfaction. Aging Ment Health 2023, 1–7. 10.1080/13607863.2023.2194851 36995262

[49] Rowe LS, Doss BD, Hsueh AC, Libet J, Mitchell AE (2011). Coexisting difficulties and couple therapy outcomes: psychopathology and intimate partner violence. J Fam Psychol.

