# Supplementary material for: Spousal collaboration mediates the relation between self-rated health and depressive symptoms of Chinese older couples: an actor-partner interdependence approach
Source: BMC Geriatr. 2024 Mar 26;24:284. doi: 10.1186/s12877-024-04834-4 (PMC10964569; doi:10.1186/s12877-024-04834-4)
Supplement: Supplementary file 1 — Supplementary Material 1 [file 12877_2024_4834_MOESM1_ESM.docx]

**Supplementary Table 1. Direct Effects and Indirect Effects for the APIMeM (control for several variables)**

| Effect | Model 1^a^ | Model 2^b^ | Model 3^c^ |
| --- | --- | --- | --- |
| husbands’ Self-rated health → husbands’ Depressive symptoms | | | |
| Total | **-0.384(-0.495,** **-0.271)**  -0.021(-0.056, 0.010)  **-0.030(-0.064,** **-0.009)**  0.009(-0.008, 0.043)  **-0.363(-0.484,** **-0.243)** | **-0.382(-0.495, -0.261)**  -0.022(-0.058, 0.008)  **-0.029(-0.064,** **-0.010)**  0.007(-0.009, 0.042)  **-0.360(-0.478,** **-0.236)** | **-0.370(-0.483,** **-0.259)**  -0.021(-0.055, 0.010)  **-0.029(-0.064,** **-0.008)**  0.008(-0.009, 0.042)  **-0.349(-0.469,** **-0.232)** |
| Total indirect |  |  |  |
| Indirect (husbands’ Spousal collaboration) |  |  |  |
| Indirect (wives’ Spousal collaboration) |  |  |  |
| Direct |  |  |  |
|  | | | |
| wives’ Self-rated health → wives’ Depressive symptoms | | | |
| Total | **-0.354(-0.469,** **-0.241)**  **-0.027(-0.056,** **-0.006)**  0.000(-0.016, 0.012)  **-0.027(-0.056,** **-0.009)**  **-0.327(-0.448,** **-0.211)** | **-0.355(-0.467, -0.244)**  **-0.029(-0.059, -0.008)**  -0.002(-0.024, 0.004)  **-0.027(-0.058, -0.009)**  **-0.327(-0.443, -0.214)** | **-0.345(-0.459,** **-0.234)**  **-0.029(-0.060, -0.008)**  -0.003(-0.027, 0.005)  **-0.026(-0.056,** **-0.008)**  **-0.316(-0.438,** **-0.202)** |
| Total indirect |  |  |  |
| Indirect (husbands’ Spousal collaboration) |  |  |  |
| Indirect (wives’ Spousal collaboration) |  |  |  |
| Direct |  |  |  |
|  |  |  |  |
| husbands’ Self-rated health → wives’ Depressive symptoms | | | |
| Total | **-0.165(-0.303,** **-0.029)**  -0.021(-0.067, 0.017)  0.001(-0.030,0.030)  **-0.021(-0.054, -0.002)**  **-0.144(-0.278, -0.010)** | **-0.124(-0.251, -0.005)**  -0.014(-0.056, 0.020)  0.007(-0.016, 0.034)  **-0.021(-0.058, -0.003)**  -0.110(-0.239, 0.017) | **-0.133(-0.271,** **-0.003)**  -0.012(-0.055, 0.025)  0.009(-0.018, 0.038)  **-0.021(-0.055, -0.002)**  -0.121(-0.253, 0.006) |
| Total indirect |  |  |  |
| Indirect (husbands’ Spousal collaboration) |  |  |  |
| Indirect (wives’ Spousal collaboration) |  |  |  |
| Direct |  |  |  |
|  | | | |
| wives’ Self-rated health → husbands’ Depressive symptoms | | | |
| Total | -0.027(-0.206, 0.142)  0.020(-0.012, 0.058)  0.009(-0.008, 0.037)  0.011(-0.013, 0.042)  -0.047(-0.222, 0.119) | -0.029(-0.213, 0.139)  0.018(-0.012, 0.059)  0.009(-0.007, 0.036)  0.009(-0.015, 0.041)  -0.047(-0.227,0.120) | -0.027(-0.211, 0.140)  0.018(-0.012, 0.057)  0.008(-0.007, 0.037)  0.010(-0.014, 0.041)  -0.045(-0.227, 0.122) |
| Total indirect |  |  |  |
| Indirect (husbands’ Spousal collaboration) |  |  |  |
| Indirect (wives’ Spousal collaboration) |  |  |  |
| Direct |  |  |  |

Note. SE Standard Error. The standardized coefficients are reported in supplementary table. Significant coefficients are in bold (The 95% confidence interval did not include 0).

^a^ model for deleting couples with anyone who is not working (N=346)

^b^ model control for educational level, number of children, age and physical limitations

^c^ model control for sample site

**Supplementary Figure 1. A conceptual framework for the relationships between health appraisal, spousal collaboration, and health outcomes**

**Health**

**APPRISAL**

**Health**

**Outcomes**

**Both Facing Physical Functioning Limitations**

**Dyadic coping process**

**Health**

**Outcomes**

**Health**

**APPRISAL**

*Notes: The black solid line represents the actor direct effect. The black dashed line represents the partner direct effect. The blue solid line represents the actor indirect effect. The red dashed line represents the partner indirect effect.*

| mediation effect pathways | R^2^ |
| --- | --- |
| husbands’ Self-rated health →husbands’ Spousal collaboration→ husbands’ Depressive symptoms | .423 |
| husbands’ Self-rated health → wives’ Spousal collaboration→ husbands’ Depressive symptoms | .127 |
| wives’ Self-rated health →husbands’ Spousal collaboration→ wives’ Depressive symptoms | .032 |
| wives’ Self-rated health →wives’ Spousal collaboration→ wives’ Depressive symptoms | .049 |
| husbands’ Self-rated health →husbands’ Spousal collaboration→ wives’ Depressive symptoms | .185 |
| husbands’ Self-rated health →wives’ Spousal collaboration→ wives’ Depressive symptoms | .407 |
| wives’ Self-rated health →husbands’ Spousal collaboration→ husbands’ Depressive symptoms | .045 |
| wives’ Self-rated health →wives’ Spousal collaboration→ husbands’ Depressive symptoms | 035 |

**Supplementary Table 2. Effect-size for the mediation effect**
